# Cranberry Supplements for Urinary Tract Infection Prophylaxis in Pregnant Women: A Systematic Review of Clinical Trials and Observational Studies on Efficacy, Acceptability, Outcomes Measurement Methods, and Studies’ Feasibility

**DOI:** 10.7759/cureus.46738

**Published:** 2023-10-09

**Authors:** Zoryana Bolgarina, Luis Fernando Gonzalez-Gonzalez, Guillermo Villamizar Rodroiguez, Alejandro Camacho

**Affiliations:** 1 Principles and Practice of Clinical Research, Harvard T.H. Chan School of Public Health, Boston, USA; 2 Obstetrics and Gynecology, California Institute of Behavioral Neurosciences and Psychology, Fairfield, USA

**Keywords:** systematic review, efficacy, side effects, safety, acceptability, feasibility, urinary tract infection, cranberry, pregnancy

## Abstract

Cranberry supplements are commonly used to prevent urinary tract infections (UTIs). However, their usefulness is uncertain in pregnant women. We aimed to comprehensively summarize the current knowledge on cranberry supplements' efficacy and acceptability during pregnancy in addition to the outcomes measurement methods and studies' feasibility. To achieve it, we searched PubMed, PMC, and Europe PMC databases plus screened citations followed by critical appraisal of included eligible English-written primary studies that (1) focused on pregnant women supplemented with any cranberry supplements; (2) provided data on cranberry supplements' efficacy, acceptability, outcomes measurement methods, and studies' feasibility; (3) included human subjects; and (4) published worldwide. Two randomized clinical trials (RCTs) and one nested cohort study, including 1156 pregnant women in total, contributed to our analysis. A tendency toward UTI reduction was demonstrated, although the results' validity was impacted by significant juice-induced gastrointestinal intolerance (23%; 44 of 188 subjects). Changing the form of supplementation from cranberry juice to capsules reduced the issue, causing side effects in one of 49 subjects (2%). Nevertheless, both RCTs still experienced significant recruitment and retention problems, which were at 33% and 59% on average, respectively. Newly acquired safety data on 919 more subjects suggests no increased risks of all malformations, vaginal bleeding, and neonatal complications. Investigating cranberry capsules' efficacy as a non-antibacterial option for UTI prevention in pregnant women has become a feasible and important direction with the current advancement in understanding cranberry supplements' actions, recommended doses plus regimens, and their safety in the population. We reviewed the challenges and discovered knowledge gaps and the implementation strategies for future studies.

## Introduction and background

Urinary tract infections (UTIs) are a frequent complication with an overall prevalence of 8% during pregnancy [1,2]. These infections include asymptomatic bacteriuria (ASB), acute cystitis, and acute pyelonephritis, affecting 2-10%, 1-4%, and 1-2% of pregnant women, respectively [3-6]. Although ASB does not require treatment in the general population, it occupies a special place in the management during pregnancy. As shown, 30-50% of untreated ASB progress to acute cystitis and/or pyelonephritis [7-8]. In turn, pyelonephritis can lead to significant maternal and neonatal complications, such as preterm birth and low birth weight babies [9]. Therefore, all UTIs during pregnancy are initially classified as complicated, requiring additional considerations. 

According to the recently published Clinical Consensus document on the management of UTIs during pregnancy by the American College of Obstetricians and Gynecologists, (1) all women should be screened once for ASB early in pregnancy; (2) any UTIs should be treated with a five to seven days course of targeted antibiotics; and (3) postcoital or continuous suppressive antibiotic treatment can be considered in women with recurrent UTIs, defined as the occurrence of at least two UTIs during pregnancy [10]. However, evidence for long-term antibiotic prophylaxis efficacy in preventing complications is limited, while inappropriate antibiotic use poses risks to developing antimicrobial resistance and adverse effects [11,12]. Therefore, the utmost interest is discovering effective and safe non-pharmacological strategies for UTI prevention in pregnant women, which are currently not accepted and recommended by the Consensus.

Cranberries are a popular food product thought to be effective in the prevention of UTI due to their phenolic compounds and A-type proanthocyanidins' (PACs) ability to hinder the adhesion of the uropathogenic bacteria [13-15]. It is thought to cause the inhibition of type 1 and type P fimbriae of *Escherichia coli*, which are responsible for the pathogenesis of cystitis and pyelonephritis, respectively [16-18]. This mechanism of action acquires particular importance since *E. coli* causes 82.5% of pyelonephritis in pregnant women [19]. Besides inhibition of adhesion, cranberry components significantly reduced beta-lactamases and other virulence gene expression in vitro, which seems to be interesting as the high levels of extended-spectrum β-lactamases are the main reason for the resistance of* E. coli* [20-22].

In addition to cranberry metabolites' activity against *E. coli*, they also showed antibacterial activity against other most common uropathogens, such as *Enterococcus faecalis*, *Staphylococcus* species, and even *Pseudomonas aeruginosa* [23-25]. Besides, they possess other functions, such as stimulating the kidney's innate immune response by induction of the Tamm-Horsfall protein, suppressing inflammatory cascade, acidifying urine, and normalizing gut microbiota, especially in subjects on a regular diet [26-30].

Recently, the Cochrane Group released a fifth update of the review on the efficacy of cranberry use in UTI prevention [31]. They concluded that supplementation with cranberries reduces the overall risk of UTIs by 30% in both genders and symptomatic infections in non-pregnant women with a history of recurrent UTIs by 26%. However, minimal research with uncertain benefits of cranberry supplements' efficacy and safety has been published on pregnant women [31]. 

Considering the described benefits of cranberries, minimal knowledge of non-antibacterial prevention during pregnancy, and lately established differences in cranberry supplements' efficacy to prevent UTIs in non-pregnant and pregnant populations, we aimed to review the full spectrum of primary clinical studies of cranberry supplements in preventing UTI during pregnancy to (1) analyze its efficacy, acceptability, outcomes measurement methods, and studies' feasibility and (2) determine the challenges and ways these issues can be addressed in future studies.

## Review

Methods

The current review followed the recommendations of the Preferred Reporting Items for Systematic Reviews and Meta-Analyses (PRISMA) [32].

Eligibility Criteria

This study focused on the inclusion of clinical trials and observational studies in which pregnant women were supplemented with nutritional cranberry supplements. We did not restrict a comparison group in any way. For the outcomes, we choose efficacy (estimates of UTI prevention ability), tolerance (any side effects or events, any safety issues related to pregnancy complications and/or neonatal outcomes, and compliance issues), studies' feasibility (recruitment rates, recruitment failure reasons, retention rates, and dropout reasons), and studies' methodology (types of studies and data collection methods). We focused on retrievable English-written primary studies that were published worldwide and excluded non-primary studies after the completion of the snowballing process for additional potentially relevant articles. We did not screen databases for unpublished articles.

Search Strategy

A systematic search was performed in Pubmed, PubMed Central (PMC), and Europe PMC databases to retrieve publications from the date of their uploading to the databases until August 2, 2023. The search strategy for each database is detailed in Appendices (Table 4).

*Studies Selection and* *Data Extraction*

**Figure 1:**
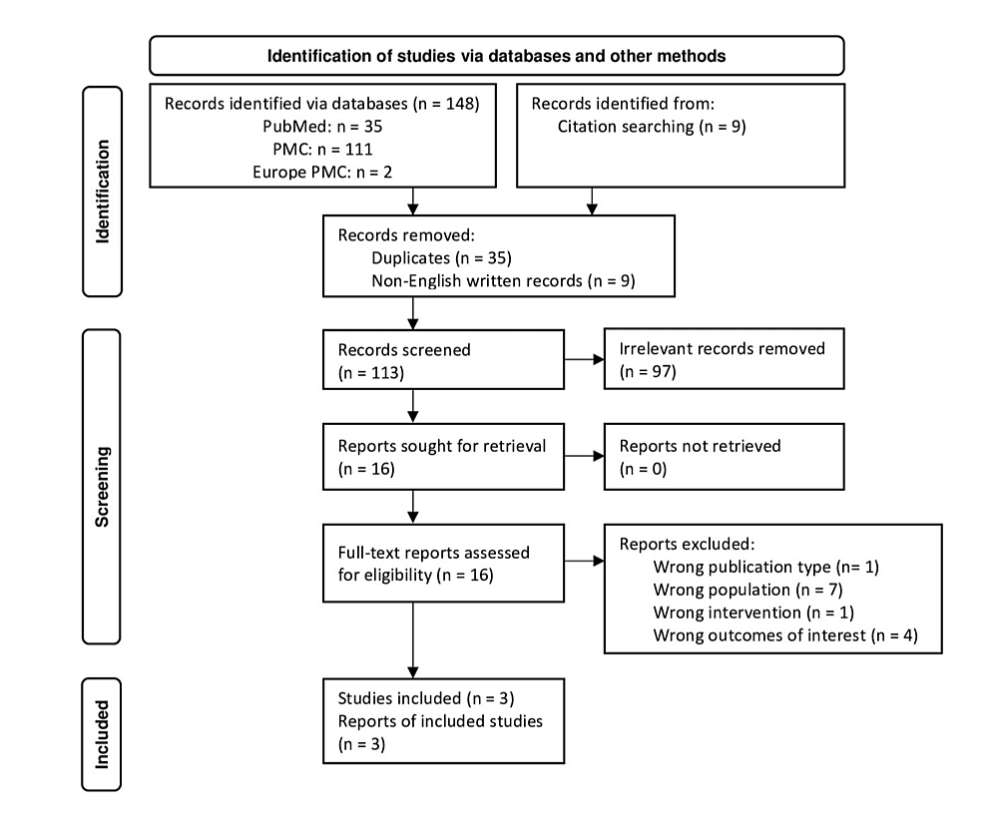
PRISMA flow diagram PRISMA, Preferred Reporting Items for Systematic Reviews and Meta-Analysis; PMC, PubMed Central

The screening process was independently hand-performed by three authors (ZB, LFGG, and JGBV), and another author (AAM) settled disagreements. After removing duplicates and non-English-written articles, the studies' eligibility was first screened by title and abstracts, and then all full-text studies that satisfied the study criteria were selected. We also reviewed the record's reference lists. The entire process is shown in Figure 1.

After retrieving eligible studies, three reviewers (AAM, GVR, and AC) independently critically appraised the studies' methodology and categorized the risk of bias for each study according to the Joanna Briggs Institute (JBI) critical appraisal tools, as follows: ≤49% ("high"), 50-69% ("moderate"), and >70% ("low") [33-36]. The studies' risk of bias assessment results and the Joanna Briggs Institute (JBI) critical appraisal tools' checklists are provided in Appendices (Tables 5-8).

We did not prepare the protocol or register the review due to the preplanned short timeline for the review completion. The data was extracted to the Excel (Microsoft Corporation, Redmond, WA, USA) file by three independent reviewers (AAM, ZB, and LFGG), including author, year, country, type of study, studies' design, sample size, entry criteria, treatment arms, products, single dose of active intervention, duration of treatment, and previously mentioned outcomes. The results are provided in Tables 1-2.

**Table 1:** RCT on cranberry products supplementation for UTI prophylaxis during pregnancy *The frequency of intervention was changed to twice daily (breakfast, dinner) after enrolling 52 of 188 subjects due to non-compliance issue PACs, proanthocyanidins; RCT, randomized controlled trial; ASB, asymptomatic bacteriuria; UC, urine culture; WG, weeks of gestation; DM, diabetes mellitus; SCD, sickle cell disease; HTN, hypertension; TID, trice daily (ter in die); OD, once daily (omne in die); BID, twice daily (bis in die); UTI, urinary tract Infection; CFU, colony forming units; LBW, low birth weight; NICU: neonatal intensive care unit; LE, leukocyte esterase; UA, urinalysis; C&S, culture and sensitivity; IRR, incidence risk ratio; N/V, nausea/vomiting; IQR, interquartile range; GI, gastrointestinal; GDM, gestational diabetes mellitus

Study	Type of study/design/sample size	Entry criteria	Arms (number of women)/product	Single dose/duration of treatment	Selected outcomes	Outcomes measurement method	Results	Notes
Wing et al. (2008) [51]	RCT (pilot)/parallel/188	Inclusion criteria	1: Vaccinium macrocarpon (Aiton) TID* (58 subjects)	240 mL of juice (106 mg of PACs)	Number of bacteriuria cases (UC: ≥100.000 CFU/mL of a single pathogen)	Monthly screening with urine dipstick	ASB	Recruitment (2005-2007)
		1. Pregnant women	2: Vaccinium macrocarpon (Aiton) OD + placebo BID (67 subjects)	Until delivery, regardless of UTI episodes		If LE & nitrites positive, then UA microscopy plus C&S	1: IRR 0.43 (0.14–1.39)	62% (313 of 501) of eligible subjects refused to participate in the trial
		2. Screened for ASB with UC before 16WG	3: Placebo TID (63 subjects)				2: IRR 0.85 (0.34–2.08)	Retention
		Exclusion criteria	Product: 27% juice provided by Fisher BioServices Corporation in partnership with Ocean Spray Cranberries, Inc.™				3: IRR 1.0	38.8% (73 of 188) of subjects withdrew from the trial
		1. DM					Any UTIs:	60% (44 of 73) of the losses related to side effects
		2. SCD					1: IRR 0.59 (0.22–1.60)	
		3. Chronic HTN					2: IRR 0.85 (0.36–2.01)	
		4. Kidney failure					3: IRR 1.0	
		5. Chronic renal disease			Side effects	Dietary diary	N/V, diarrhea	
		6. Known urologic abnormalities					Taste intolerance	
		7. Antimicrobial therapy at/within two weeks of screening			Preterm birth <37WG and <34WG	Events	No statistically significant difference (113 of 125 women)	
					Route of delivery			
					LBW			
					Apgar <7 at 1 minutes & <9 at 5 minutes			
									Admission to NICU
					Compliance				Dietary diary (labels from bottles) plus a self-reported percentage of compliance with the dosing schedule	Overall compliance with any daily dose:
						1: 53.5% (31/58)			
						2: 61.2% (41/67)			
						3: 68.2% (43/63)			
						Median number of days in the study			
						1: 152.5 (IQR 56–183)			
						2: 158 (IQR 61–181)			
						3: 171 [IQR 76–185]			
					Wing et al. (2015) [53]	RCT (pilot)/Parallel/49	Inclusion criteria		1: Vaccinium macrocarpon (Aiton) BID (24 subjects)	Two capsules (32-34 mg of PACs)	Number of bacteriuria cases (UC: ≥100.000 CFU/mL of a single pathogen)	Monthly screening with urine dipstick	ASB	Recruitment (2009-2012)
1. Pregnant women	2: Placebo BID (25 subjects)	Until delivery, regardless of UTI episodes	If LE & nitrites positive, then UA microscopy plus C&S	1: two cases			78% (174 of 223) of eligible subjects refused to participate in the trial				
2. Screened for ASB with UC before 16WG	Product: TheraCran(R) product supplied by Ocean Spray Cranberries, Inc.™			2: five cases			Retention				
Exclusion criteria			Side effects	Dietary diary			GI intolerance (no statistically significant difference)	20% (10 of 49 women) withdrew from the trial before 1st follow-up visit due to interest loss
1. DM							1: 81% (13/16)	Plus 29% (14 of 49 women) were lost due to
2. SCD							2: 55% (12/22)	1. Insurance restriction (nine cases)
3. Chronic HTN			Preterm birth <37WG	Events			No statistically significant difference (14 of 24 women)	2. Loss of interest (four cases)
4. Kidney failure			Route of delivery					3. Loss of appetite (one of 49 women; 2%)
5. Chronic renal disease			LBW <2500 grams					
6. Known urologic abnormalities			Apgar <7 at 1 minutes & <9 at 5 minutes					
7. Antimicrobial therapy at/within two weeks of screening			Admission to NICU					
			Compliance	Capsule counts plus a self-reported percentage of compliance with the dosing schedule			Overall compliance	
							1: 69% (11/16)	
							2: 77% (17/22)	
							Compliance data (%) availability with duration of the study	
							1st month: 74%	
							2nd month: 71%	
							3rd month: 67%	
							4th months 65%	
							5th month: 41%	
							6th month: 8%	

**Table 2:** Observational studies on cranberry supplementation for UTI prophylaxis during pregnancy MBRN: Medical Birth Registry of Norway; WG: weeks of gestation; VB: vaginal bleeding; BW: birth weight; LBW: low birth weight; SGA: small for gestational age; ICD: International Classification of Diseases; w/o: without; SCM: sternocleidomastoid muscle *MoBa is the Norwegian mother and child population-based prospective cohort study

Study	Design/sample size	Entry criteria	Exposure/regimen	Data collection	Selected outcomes	Results
Heitmann et al. (2013) [52]	Nested prospective cohort study	Inclusion criteria	Cranberry use	1st questionnaire at 13-17WG PLUS routine fetal US at 17-18WG	VB <17WG & ≥17WG	919 exposed subjects
	Sample size: 68.522	1. MoBa study's participants (available MBRN records plus 1st questionnaire results)	1. Overall	2nd questionnaire at 30WG	Hospitalization due to VB <17WG & ≥17WG	566 exposed subjects <17WG (≥80% of statistical power to rule out all malformations, serious malformations, LBW, low Apgar score, preterm birth, neonatal infections, cardiac malformations, or stillbirth/neonatal death)
		Exclusion criteria	2. Early pregnancy (<17WG)	3rd questionnaire at six months postpartum	VB more than spotting after 17WG	122 exposed women during the first trimester revealed no increased risk of defects
		1. Multiple pregnancies	3. Late pregnancy (≥17WG)		All malformations	11 infants had malformations w/o specific pattern
		2. Delivered infants had multiple birth defects	Unknown daily dose, duration of treatment, total dose, and adjunct herbals		Stillbirth (≥22WG or BW of ≥425 gram)	1) two cases of hypospadias
					LBW (<2500 grams)	2) two cases of macrocephaly
					SGA (<10th percentile)	3) four cases of hip or feet dislocation/deformity
					Preterm birth (<37WG)	4) one case of SCM deformity
					Apgar score <7 at 5 minutes	5) one case of ankyloglossia
					Neonatal infections (ICD-10 P35-39)	6) one case of undescended testicles
						Cranberry users more frequently experienced vaginal spotting after 17WG, but no association was found after adjustments for confounders

Data Synthesis

We pooled continuous variables by sum dividing by the total to obtain average data. The characteristics and results were grouped according to the categorized data and displayed in tables.

Results

Studies Selection and Critical Appraisal

Our search resulted in 157 publications. After removing duplicates (n=35) and non-English written records (n=9), we screened 113 titles and abstracts for relevance, ending with 16 full-text articles after a successful retrieval step. We identified 13 reports as ineligible due to the wrong publication type, wrong population, wrong intervention, and wrong outcomes of interest [37-50].

The final three selected studies underwent a critical appraisal process, scoring "low" and "moderate" risk of bias [51-53]. The last study was a pilot trial with a small sample size evaluating subjects' compliance with cranberry capsule use.

Studies Characteristics

This review included two pilot randomized controlled trials (RCTs) and one observational study [51-53]. The same authors conducted the RCTs in the USA, while another author performed the cohort study in Norway. RCTs collected data on the studies' feasibility, all three studies analyzed safety, and one clinical trial evaluated efficacy [51-53]. All studies had parallel study designs.

The total number of pregnant women who ingested cranberry supplements in all studies was 1156. Both RCTs targeted 237 relatively healthy pregnant women who underwent early screening for ASB, evaluating mainly compliance with supplementation by cranberry juice and capsules [51,53]. Of those 237 women, the efficacy of cranberry juice estimation is based on the collected data from 188 subjects. In addition to RCTs participants' available safety information (127 women), the large nested cohort study within the Norwegian mother and child prospective cohort study (MoBa) analyzed the safety of cranberries supplementation by 919 pregnant women; thus, 1046 in total.

Intervention and Exposure Characteristics

The first placebo-controlled RCT compared three arms and evaluated the Vaccinium macrocarpon (Aiton) (27% juice provided by Fisher BioServices Corporation in partnership with Ocean Spray Cranberries, Inc.™) juice efficacy, given three (720 mL, containing 324 mg of PACs) and one (240 mL, containing 106 mg of PACs) times daily from the early term of gestation until delivery. However, the trial faced significant compliance and tolerability issues, requiring a dose reduction. As a result, the actual "dosing did not differ between groups" [51]. In the next similar placebo-controlled RCT, participants received two capsules of Vaccinium macrocarpon (Aiton) (TheraCran's product supplied by Ocean Spray Cranberries, Inc.™) twice daily, which is 64-68 mg of PACs in total, instead of juice [53]. The observational study did not collect information on daily doses in amount of PACs (unspecified products), treatment duration, or total cranberry metabolites exposure measurements during pregnancies; however, their study evaluated the association between exposure and all fetal malformations, vaginal bleeding (VB), and neonatal outcomes during periods of cranberry use before 17 weeks of gestation (WG), at/after 17WG, and overall [52].

Recruitment and Retention Rates

Pooled recruitment and retention rate calculations are shown in Table 3. In both RCTs, the recruitment ranged from 22% to 38% (33% on average), enrolling 16-94 participants on average per year due to refusals and dislike of cranberries’ taste [51,53]. The dropout rate was also high, reaching 39-49% (41% on average). However, the reasons behind recruitment problems were different. In the first clinical trial on supplementation with cranberry juice, 23% (44 of 188) withdrew due to side effects, while the additional 15% (29 of 188) left on an unreported basis [51]. On the other hand, women who received cranberry capsules tolerated the intervention noticeably better, with only one of 49 subjects (2%) lost for follow-up due to impaired appetite; the rest of the participants withdrew due to disinterest in participation (14 of 49; 29%) and insurance restrictions (nine of 49; 18%) [53]. Treatment adherence dropped below 74% between the first and fifth months of enrollment based on the information provided by subjects and the median days of participation in the trial [51,53].

**Table 3:** Recruitment, dropout, and retention rates in the clinical trials

	Eligible subjects	Enrolled subjects	Recruitment rates	Lost for follow-up	Dropout rates	Retention rates
Wing et al. (2008) [51]	501	188	188/501 (38%)	73	73/188 (39%)	61%
Wing et al. (2015) [53]	223	49	49/223 (22%)	24	24/49 (49%)	51%
Total	724	237	237/724 (33%)	97	97/237 (41%)	59%

Efficacy, Side Effects, and Safety

One trial reported the efficacy in terms of ASB and any UTI prevention estimates, showing a tendency toward incidence reduction [51]. Two studies collected data on adverse effects, such as gastrointestinal and taste intolerance, that were less disturbing with using cranberry capsules than juice - 2% (1 of 49) versus 23% (44 of 188) of participants [51,53]. All three studies collected data on safety inclusive of preterm birth, route of delivery, low birth weight, Apgar score below seven at the first minute and fifth minutes, admission to NICU, VB before 17WG and at/after 17WG, hospitalization due to VB before 17WG and at/after 17WG, all malformations, stillbirth/neonatal death, small for gestational age, and neonatal infections [51-53]. Of 1046 women, 122 and 571 ingested cranberry supplements in the first trimester and between 12-to-16 WG, respectively. Among those 571 participants, 444 subjects’ data contributed to the malformation risk assessment. Accumulated data did not find increased safety risks along with any specific patterns of malformations.

Discussion

This is the first systematic review that comprehensively analyzes the determinants of intervention implementation strategies of supplementation with cranberry supplements during pregnancy.

Recruitment Capability and Sample Characteristics

Historically, the recruitment of pregnant women is a particular challenge. Both included RCTs did not escape from being able to enroll only 33% of 724 eligible women (13% of 1858 screened participants passing broad eligibility criteria focused on healthy pregnant women), which was notably low compared to the expected overall interest at an 86% rate of pregnant women to participate in trials investigating "healthy eating" interventions [54]. The authors reported restricted information about ineligible subjects (underreported reasons), recruitment strategies (unknown), and enrollment failure reasons (of note, similar). In addition, the recruitment period took two to three years, enrolling 94 to 16 participants per year in two centers. Thus, a thoughtful approach is needed to plan this step in future studies. In addition to selecting inclusive criteria, motivated target population, and center(s) accessible to eligible members, prioritizing cooperation with local obstetricians and prenatal planning specialists described by Sutton et al. (2017) can be the first step toward success [54]. Moreover, later, we will provide a theoretical explanation of possible dropout prevention due to participants' disbelief in treatment or suggestion that they have been assigned to a placebo arm by informing women that they would still experience UTI episodes, regardless of their allocation.

Data Collection and Outcome Measures

Ensuring medication adherence and selecting suitable outcome measurements are necessary to achieve adequate power and obtain valid results. As shown, "sloppy" compliance of 60-89% contributed by 30-70% may lead to efficacy result miscalculation [55]. However, achieving optimal compliance is challenging. For instance, Olesen et al. (2001) discovered that only 43% of pregnant women adhere to their medication regimen [56]. Wing et al. (2015) also detected a downtrend in compliance with cranberry capsule ingestion that reached below the 65% level at the end of the fourth month [53]. 

In choosing the compliance assessment tool, it is known that there is no ideal and simultaneously accurate and inexpensive measurement method. Analyzed studies relied on subjective (a dietary diary's information, subject's self-estimation) and objective (capsule counts) approaches, which are simple and suitable although less accurate, carrying the risk of compliance overestimation [51,53,57,58]. Apart from these methods, selecting a motivated population interested in intervention benefits, sharing decision-making, simplifying dosing demand, monthly enchanting of subjects-provider (researcher) collaboration building trustful relationships and satisfied experience, proactively addressing pregnant women's concerns, and applying other strategies summarized by Spilker's (1992) and Parks et al. (2022) articles' tables may be supportive aids [59-66].

To evaluate the efficacy of cranberry juice, Wing et al. (2015) tested participants with a urinary dipstick, a routine test used during monthly antenatal visits [53]. Although it reflects real practice, it is insufficient and less sensitive to detect ASB (still feasible and acceptable) and does not eliminate the burden of additional evaluation (urine cultures), which can be misperceived by subjects as study-related and, consequently, compromising the retention rate [67,68]. Simplified monthly screening for ASB with a midstream urine culture could benefit subjects, leading to accurate estimation of the result and prevention of a serious "increased diagnostic burden"-associated dropout rate.

Acceptability and Suitability

Previous studies revealed that cranberry supplements' most bothersome side effect is gastrointestinal distress, which seems worse when using juice and becomes unacceptable with increasing juice daily amounts - 23% of pregnant women left the study [51]. As expected, delivering the active components in capsule form did not lead to statistically significant gastrointestinal disturbances compared to a placebo group in pregnant women [53]. Besides, it seems an appealing diet intervention to pregnant women, considering no losses for follow-up due to moderate-to-severe side effects and four-month participants' compliance above the average level of 43% determined by Olesen et al. (2001) [56]. The findings are also congruent with the mentioned general pregnant women's interest in "healthy diet" interventions.

Since the last enrolled participant in the RCT performed by Wing et al. (2015), a new nested cohort within the prospective cohort study remarkably extended the current knowledge on the safety of cranberry supplements usage by 919 pregnant women [52,53]. Of note, Olesen et al. (2001) showed that the main concern of pregnant women's non-adherence to medication seems to be fetal safety [56]. Another study supported this concern, revealing pregnant women's tendency to overestimate malformation risk in 96% of cases [69]. Although none of the dropped-out participants in Wings et al. trials (2008; 2015) pointed to safety concerns, the accumulated new data would likely improve recruitment and retention rates [51,53]. As for the maternity risks, no meta-analyses, systematic reviews, RCTs, or observational studies have reported any severe adverse events.

Intervention and Participant Responses

Earlier, we mentioned the estimated overall cranberry supplement efficacy in UTI reduction of 30% [31]. Though it is barely investigated in pregnant women, preliminary findings have shown a promising tendency [37,51]. However, these trials encountered a significant data loss predominantly due to gastrointestinal dysfunction (39-56%), which is unlikely could be handled effectively. The feasible solution seems to be supplementation with cranberry capsules instead of juice - as shown by Wing et al. (2015), the excellent daily tolerability of the capsules contained 64-68 mg of PACs [53].

To our knowledge, no studies have explored the effective dosage regimen of cranberry supplements in pregnant women. In nonpregnant women with normal kidney function, Howell et al. (2010) established dose- and time-dependent efficacy of 36 and 72 mg of PACs daily dosages, stating that 36 mg taken twice daily is more beneficial because (1) single 72 mg of PACs administration preserves significantly lower adhesion index after 24 hours, and (2) splitting the dose in two may provide better protective coverage as the PACs activity's peak occurs in six hours post-ingestion after any of the dosages [40]. Reviewing studies listed in Cochrane's review, we think that the key to detecting the change in an outcome is (1) a theoretical aligning of a population of interest with the dosage of cranberry supplements containing soluble PACs and the outcome measures plus (2) control for important confounders (e.g., time of treatment with antibiotics, bladder dysfunction in older women), which suffer across trials [31]. For example, pregnant women with recurrent UTIs might benefit from 72 mg of PACs compared to those at low risk for pregnancy complications. Besides, the selection of a lower dose of PACs requires special attention in the designing stage of a trial as it will be more sensitive to deviation from the intervention (e.g., poor compliance) and retention issues.

In electing suitable outcome measures, we think it is essential to consider the population of interest and the follow-up period's duration, as cranberry metabolites impact bacterial virulence factors but do not kill them [23]. For instance, nonpregnant women with a history of at least two UTI events within 12 months is one of the most frequently studied populations. At the same time, the six-month risk of UTI recurrence is 24% after a single UTI [70]. Thus, measuring the efficacy by prevention of "at least one UTI event" in proportions would require the assumption of cranberry's ability to suppress bacterial colonization for at least six months, which is possible, but (1) it would require a large sample size, plus (2) careful considerations of local population characteristics and tendencies to obtain predictable results. Still, it requires a backup outcome measurement from an ethical perspective. Therefore, the most appropriate outcomes should account for UTI's opportunity to cluster in time, which can be the difference in the total or, to improve the studies' generalizability and comparability, the incidence density of UTI events between groups [71]. For this goal, we refer to Maki's et al. (2016) study as exemplary in applying these concepts [71].

Strengths and Limitations

The study endows the strengths of a systematic review that answered a narrow research question on the cranberry supplements' efficacy, acceptability, outcomes measurement methods, and the studies' feasibility parameters during pregnancy. In light of the Cochrane review published a few months ago, our search strategy was comprehensive and, as a result, indirectly covered the availability of non-English-written literature on the topic [31]. Besides, we screened a European database and included observational studies, which is especially important for accumulating data on side effects/events and safety. However, search bias is still possible, as we limited our search strategy to three databases and avoided unpublished databases or could not include studies of developing countries with poorly organized studies uploading processes to well-known databases. Considering our large team, we spent a lot of time and had plenty of discussions during the study selection, critical appraisal process, and data extraction step. We also had intense brainstorming of the performed studies' challenges and possible solutions. Among limitations, our results are based on the quantity (e.g., small number of published studies on the topic) and quality data (e.g., poor methodological quality of included studies due to unacceptable level of missing data, heterogeneity of the studies in terms of type of product, dosage, frequency of administration, etc.) obtained predominantly by two groups of researchers.

## Conclusions

We explored the current knowledge of supplementation with cranberry supplements to prevent UTIs during pregnancy. Overall, the results showed this is a promising but under-investigated direction, likely due to feasibility issues and an extended follow-up period. Among the obstacles, we identified a low average recruitment rate of 33% and a substantial average dropout rate of 41% (resulting in unacceptable missing data levels to obtain reliable estimation of their prophylactic effect in pregnant women). Newly generated knowledge on a better understanding of the cranberry's mechanism of action and effective regimens has become sufficient to overcome the theoretical connection between selected populations and outcomes. In addition, accumulated data on cranberry supplements' malformation risks makes the investigation more feasible and ethical, although it demands measured dose-dependent confirmation as a secondary outcome.

In general, considerable evidence supports the connection between cranberry supplement consumption and its ability to prevent UTI recurrence. Nonetheless, this field of knowledge exploration acquired some chaotic features in terms of urine culture threshold differences in defining clinical UTI, unstandardized products based on reliably measured amounts of PACs inside them, unexplained choice of the regimens, and theoretical misalignment between the purposes and selected measured outcomes, overwhelming databases with conflicting results. Thus, based on our example, we call other researchers to organize the area contingent on populations, standardized cranberry supplements' dosages, and measured outcomes of interest. In addition, performing a systematic review or meta-analysis on risk factors for UTI recurrence is valuable for identifying confounders that need to be controlled. As for pregnant women, focusing on motivated populations, selection of cranberry supplements in the form of capsules, supplementing women with at least 72 mg of PACs daily, and estimation of the prophylactic effect by detecting the difference in the incidence density of UTI events between groups, along with the introduction of the discussed intervention implementation strategies, might finalize in the first high-quality clinical trial to answer the research question on cranberry supplements' efficacy in pregnant women and, in the case of success, spread up future investigations (e.g., dose selection indications). Besides, an update on the prevalence of UTI and associated pregnancy complications is desirable.

## Appendices

**Table 4:** Database search requests

Database	Search query	Results
PubMed	(("Pregnancy"[Mesh] OR "Pregnant Women"[Mesh] OR "pregnancy"[Text Word] OR "pregnant"[Text Word]) AND ("Vaccinium macrocarpon"[Mesh] OR "Viburnum"[Mesh]OR "Proanthocyanidins"[Mesh] OR "vaccinium macrocarpum"[Text Word] OR "viburnum"[Text Word] OR "cranberr*"[Text Word] OR "proanthocyanidins"[Text Word])) AND ("Bacteriuria"[Mesh] OR "Urinary Tract Infections"[Mesh] OR "asymptomatic bacteriuria"[Text Word] OR "bacteriuria"[Text Word] OR "cystitis"[Text Word] OR "pyelonephritis"[Text Word])	35
PMC	((((((("pregnancy"[MeSH Terms]) OR "pregnant women"[MeSH Terms]) OR "pregnancy"[Text Word]) OR "pregnant"[Text Word]))) AND (((((((("vaccinium macrocarpon"[MeSH Terms]) OR "viburnum"[MeSH Terms]) OR "proanthocyanidins"[MeSH Terms]) OR "vaccinium macrocarpum"[Text Word]) OR "viburnum"[Text Word]) OR "cranberr*"[Text Word]) OR "proanthocyanidins"[Text Word]))) AND (((((("bacteriuria"[MeSH Terms]) OR "urinary tract infections"[MeSH Terms]) OR "asymptomatic bacteriuria"[Text Word]) OR "bacteriuria"[Text Word]) OR "cystitis"[Text Word]) OR "pyelonephritis"[Text Word])	111
Europe PMC	(KW:"pregnancy" OR KW:"pregnant women" OR "pregnancy" OR "pregnant") AND (KW:"vaccinium macrocarpon" OR KW:"viburnum" OR KW:"proanthocyanidins" OR "vaccinium macrocarpum" OR "viburnum" OR "cranberr*" AND "proanthocyanidins") AND (KW:"bacteriuria" OR KW:"urinary tract infections" OR "asymptomatic bacteriuria" OR "bacteriuria" OR "cystitis" OR "pyelonephritis")	2

**Table 5:** Critical appraisal of the included randomized clinical trials Q, question; Unk, unknown

Study	Q1	Q2	Q3	Q4	Q5	Q6	Q7	Q8	Q9	Q10	Q11	Q12	Q13	Score
Wing et al. (2008) [49]	Yes	Unk	Yes	Yes	Yes	Yes	Unk	Yes	Yes	No	Yes	Yes	Yes	77%
Wing et al. (2015) [51]	Yes	Unk	No	Yes	Yes	Yes	Unk	Yes	Yes	No	Yes	Yes	Yes	69%

**Table 6:** Critical appraisal of the included observational study Q, question; Unk, unknown

Study	Q1	Q2	Q3	Q4	Q5	Q6	Q7	Q8	Q9	Q10	Q11	Score
Heitmann et al. (2013) [50]	Unk	Yes	Yes	Yes	Yes	Yes	Yes	Yes	Yes	No	Yes	82%

**Table 7:** The Joanna Briggs Institute checklist for critical appraisal of randomized controlled trials

Abbreviation	Question
Q1	Was true randomization used for the assignment of participants to treatment groups?
Q2	Was allocation to treatment groups concealed?
Q3	Were treatment groups similar at the baseline?
Q4	Were participants blind to treatment assignment?
Q5	Were those delivering the treatment blind to treatment assignment?
Q6	Were treatment groups treated identically other than the intervention of interest?
Q7	Were outcome assessors blind to treatment assignment?
Q8	Were outcomes measured in the same way for treatment groups?
Q9	Were outcomes measured in a reliable way?
Q10	Was follow-up complete and if not, were differences between groups in terms of their follow-up adequately described and analyzed?
Q11	Were participants analyzed in the groups to which they were randomized?
Q12	Was appropriate statistical analysis used?
Q13	Was the trial design appropriate and any deviations from the standard RCT design (individual randomization, parallel groups) accounted for in the conduct and analysis of the trial?

**Table 8:** The Joanna Briggs Institute checklist for critical appraisal for cohort studies

Abbreviation	Question
Q1	Were the two groups similar and recruited from the same population?
Q2	Were the exposures measured similarly to assign people to both exposed and unexposed groups?
Q3	Was the exposure measured in a valid and reliable way?
Q4	Were confounding factors identified?
Q5	Were strategies to deal with confounding factors stated?
Q6	Were the groups/participants free of the outcome at the start of the study (or at the moment of exposure)?
Q7	Were the outcomes measured in a valid and reliable way?
Q8	Was the follow-up time reported and sufficient to be long enough for outcomes to occur?
Q9	Was follow-up complete, and if not, were the reasons for the loss to follow up described and explored?
Q10	Were strategies to address incomplete follow up utilized?
Q11	Was appropriate statistical analysis used?
