# Correction: Cranberry Supplements for Urinary Tract Infection Prophylaxis in Pregnant Women: A Systematic Review of Clinical Trials and Observational Studies on Efficacy, Acceptability, Outcomes Measurement Methods, and Studies’ Feasibility

**DOI:** 10.7759/cureus.c140

**Published:** 2023-10-16

**Authors:** Zoryana Bolgarina, Luis Fernando Gonzalez-Gonzalez, Guillermo Villamizar Rodroiguez, Alejandro Camacho

**Affiliations:** 1 Principles and Practice of Clinical Research, Harvard T.H. Chan School of Public Health, Boston, USA; 2 Obstetrics and Gynecology, California Institute of Behavioral Neurosciences and Psychology, Fairfield, USA

This article has been corrected at the request of the authors to remove Audrey A. Merriam and Jose Guillermo Betancourt-Villalobos from the author list. As stated to the journal, it has been determined that the contributions of these Dr. Merriam and Dr. Betancourt-Villalobos did not warrant authorship. All authors are in agreement regarding this decision.

